# Dimension and Location of the Mandibular Lingula: Comparisons of Gender and Skeletal Patterns Using Cone-Beam Computed Tomography

**DOI:** 10.1155/2020/2571534

**Published:** 2020-02-13

**Authors:** Kun-Jung Hsu, Yu-Chuan Tseng, Shih-Wei Liang, Szu-Yu Hsiao, Chun-Ming Chen

**Affiliations:** ^1^Graduate Institute of Dental Sciences, School of Dental Medicine, Kaohsiung Medical University, Kaohsiung, Taiwan; ^2^Department of Dentistry, Kaohsiung Municipal Ta-Tung Hospital, Kaohsiung, Taiwan; ^3^Department of Orthodontics, Kaohsiung Medical University Hospital, Kaohsiung, Taiwan; ^4^Department of Dentistry for Child and Special Needs, Kaohsiung Medical University Hospital, Kaohsiung, Taiwan; ^5^Department of Oral and Maxillofacial Surgery, Kaohsiung Medical University Hospital, Kaohsiung Medical University, Kaohsiung, Taiwan

## Abstract

**Objective:**

The study aimed to investigate and measure the anatomical relationship between the mandibular lingula (Li) and skeletal patterns using cone-beam computed tomography (CBCT). *Materials and Methods*. In total, 72 participants (23 men and 49 women) were categorized into three groups according to their skeletal patterns (specifically, the A point–nasion–B point (ANB]) angle) as follows: Class I (0° < ANB < 4°), Class II (ANB ≥ 4°), and Class III (ANB ≤ 0°). The CBCT images of 144 rami were collected, and the distance from the Li to the external oblique ridge (Li-EOR), internal oblique ridge (Li-IOR), posterior border of the ramus (Li-PBR), inferior border of the ramus (Li-IBR), sigmoid notch (Li-Sm), and mandibular foramen (Li-MF) was examined. The Li-hMF (horizontal distance from the Li to the MF) and Li-vMF (vertical distance from the Li to the MF) were measured. The comparisons of gender, side (right and left), and skeletal patterns were then evaluated by statistical analysis.

**Results:**

The values of the Li-EOR and Li-PBR (19.99 mm and 15.93 mm, respectively) were significantly higher in men than in women (18.85 mm and 14.89 mm, respectively). Moreover, the Li-IBR was higher in men (32.91 mm) than in women (30.40 mm). Both sides (right and left) and skeletal patterns demonstrated that the Li-EOR, Li-IOR, and Li-PBR were not significantly different. Pearson's correlation test reported a strong correlation between the Li-EOR and Li-IOR (*r*=0.610).

**Conclusion:**

The distances from mandibula lingula to the external oblique ridge, posterior border of ramus, and inferior border of ramus were significantly longer in men than in women. Similarly, both horizontal and vertical distances from the lingula to the mandibular foramen were significantly longer in men than in women. Therefore, the results demonstrated that the Li was longer and more protruding in men than in women. With respect to the horizontal distance from the mandibular lingula to the mandibular foramen, of the three types of the skeletal system (Class I, Class II, and Class III), Class III was the significantly largest.

## 1. Introduction

The inferior alveolar nerve (IAN) is a branch of the mandibular nerve of the fifth cranial nerve. From the mandibular foramen (MF), the IAN travels through the mandibular canal before anteriorly passing through the mental foramen toward the soft tissues of the lower lips. Because the IAN innervates the lower lip, protecting the IAN from injury during ramus surgery is the primary goal. Owing to factors such as mandibular development and muscular attachment, the MF margin can be irregular. The MF front exhibits prominent bone, the top of which presents a projection ridge, which is anatomically known as mandibular lingula (Li) [1, 2]. Since the IAN enters the MF at the Li position, an understanding of the structures surrounding the Li and MF is therefore crucial.

As surgical treatment for mandibular prognathism, the mandibular setback procedure is usually conducted using two techniques: intraoral vertical ramus osteotomy (IVRO) and sagittal split ramus osteotomy (SSRO). The procedure of IVRO extends from the sigmoid notch, vertically behind the Li and MF, to the inferior border of the mandible. The horizontal procedure of SSRO is performed above the Li and cuts through the medial cortex of the ramus. The position of the Li can determine the surgical procedures, denote injury of IAN vascular bundles, and reveal the presence of lower lip numbness. This description demonstrates that the location of the Li is very important for ramus surgery. This study investigated the Li and its relationship among three different types of skeletal patterns.

## 2. Materials and Methods

A total of 144 rami (72 patients; 23 men and 49 women) were included. The participants were further categorized into three groups according to their skeletal patterns (specifically, the A point–nasion–B point [ANB] angle): Class I (0° < ANB < 4°), Class II (ANB ≥ 4°), and Class III (ANB ≤ 0°). Based on the A point–nasion–B point angle, participants were classified into skeletal Class I (26 individuals), skeletal Class II (21 individuals), and skeletal Class III (25 individuals) according to Riedel's classification [3]. Cone-beam computed tomography (CBCT) was conducted at the Department of Dentistry, Kaohsiung Medical University Hospital. Patients with the following conditions were excluded: (1) cranial or facial cyst or tumor, (2) innate cranial or facial deformities, and (3) trauma or surgery on the cranial or facial skeleton.

**Figure 1:**
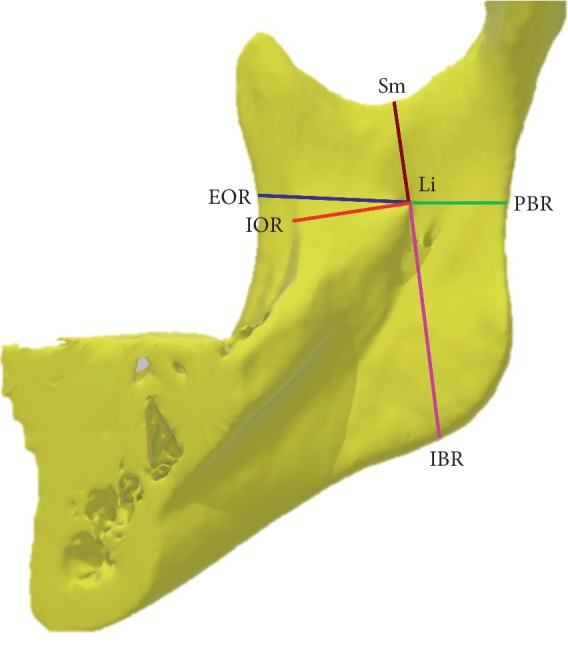
Location of the mandibular lingula. Measured reference points for lingula tip (Li). External oblique ridge (EOR), internal oblique ridge (IOR), sigmoid notch (Sm). Posterior border of ramus (PBR) and Inferior border of ramus (IBR).

**Figure 2:**
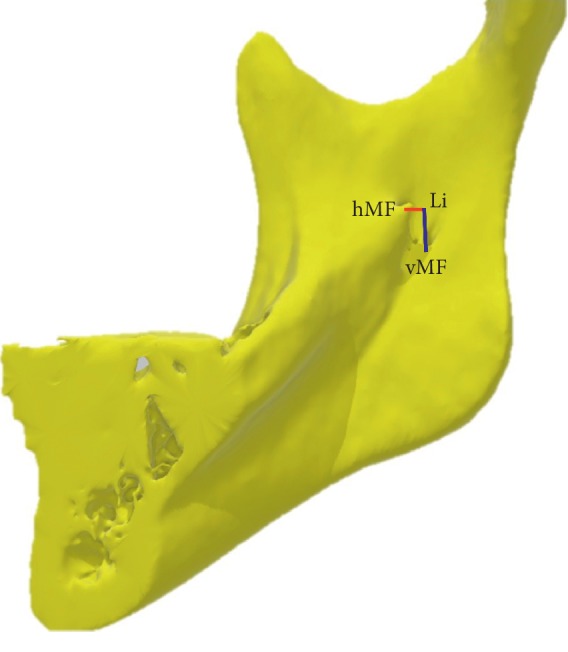
Distance between lingula and mandibular foramen. Measured reference point (mandibular foramen: MF) for lingula tip (Li). Red line (Li-hMF): horizontal distance from Li to MF. Blue line (Li-vMF): vertical distance from Li to MF.

CBCT was conducted with the calibration of three-dimensional (3D) positions on the skull. The Frankfort horizontal (FH) plane was defined as the plane connecting the right orbitale and porion on both sides. CBCT images were imported using Digital Imaging and Communications in Medicine into RadiAnt DICOM Viewer (version 4.6.9, Medixant, Poznan, Poland). The function of 3D image reconstruction was also used by extracting the ramus for 3D reconstruction. Subsequently, CBCT NNT Viewer was run to adjust the FH planes to a horizontal position. The following landmarks were identified (Figures 1 and 2): tip of the Li, external oblique ridge (EOR), internal oblique ridge (IOR), posterior border of the ramus (PBR), inferior border of the ramus (IBR), sigmoid notch (Sm), and mandibular foramen (MF). The distances were measured as follows: (1) Li-EOR: distance from the Li to the EOR, (2) Li-IOR: distance from the Li to the IOR, (3) Li-PBR: distance from the Li to the PBR, (4) Li-IBR: distance from the Li to the IBR, (5) Li-Sm: distance from the Li to the Sm, (6) Li-hMF: horizontal distance from the Li to the MF, and (7) Li-vMF: vertical distance from the Li to the MF.

Data were analyzed using IBM SPSS 20 (SPSS Inc., Chicago, IL, USA). A Student's *t*-test was conducted for continuous variables of length. Data obtained were categorized based on the side (right or left) and gender (man or woman). Intergroup comparative analysis was performed with one-way analysis of variance, and post hoc comparisons were conducted using Tukey's honestly significant difference test. Pearson's correlation was used to determine correlations between variables, whereby a *p* value of 0.05 was considered statistically significant. The strengths of correlation were described for the absolute value of the ratio of the compared variables as follows: very weak (0–0.19), weak (0.20–0.39), moderate (0.40–0.59), strong (0.60–0.79), and very strong (0.80–1.0). This retrospective research was approved by the Institutional Review Board of Kaohsiung Medical University Hospital (KMUH-IRB-20160066).

## 3. Result

Concerning the horizontal position of the Li in all participants (Table 1), the Li-EOR was 19.21 mm, Li-IOR was 12.66 mm, and Li-PBR was 15.22 mm. Based on the comparison between the left and right sides, they (Li-EOR, Li-IOR, and Li-PBR) showed no significant difference. In Table 2, the values of the Li-EOR and Li-PBR (19.99 mm and 15.93 mm, respectively) were significantly higher in men than in women (18.85 mm and 14.89 mm, respectively). Conversely, for the Li-IOR, the results obtained for men (13.20 mm) and women (12.40 mm) exhibited no significant difference. The comparison of the skeletal patterns demonstrated that the Li-EOR, Li-IOR, and Li-PBR were not significantly different. Regarding the vertical position of the Li in all participants, the Li-Sm was 20.04 mm and Li-IBR was 31.20 mm. On the basis of the comparison between the left and right sides, the Li-Sm and Li-IBR presented no significant difference. However, the Li-IBR was higher in men (32.91 mm) than in women (30.40 mm). Similarly, the Li-Sm and Li-IBR were not significantly different among the skeletal patterns.

With respect to the horizontal distance between the Li and MF (Table 1), the average Li-hMF of all participants was estimated to be 2.27 mm. Moreover, no significant difference was observed between the right and left Li-hMF values. In Table 2, the Li-hMF among men (2.81 mm) significantly exceeded that among women (2.01 mm). In Table 3, the Li-hMF of participants from Class III (2.81 mm) was significantly longer than that of those from Class II (1.80 mm), whereas the Li-hMF of Class I participants (2.12 mm) was significantly longer than that of Class II participants (1.80 mm). No significant difference was observed between Class III and Class I.

Pearson's correlation test (Table 4) reported a strong correlation between the Li-EOR and Li-IOR (*r*=0.610). Investigating the relationship between the horizontal and vertical positions of the Li, the Li-EOR, and the Li-IOR was not correlated to the Li-Sm and Li-IBR. The correlation between the Li-PBR and Li-IBR (*r*=0.338) and between the Li-IBR and Li-vMF (*r*=0.329) was significant, but weak. The Li-hMF was positive, but weakly correlated with the Li-EOR (*r*=0.278) and Li-IOR (*r*=0.329). Conversely, the Li-hMF was negative but weakly correlated with the Li-PBR (*r*=−0.205) and Li-IBR (*r*=−0.235).

## 4. Discussion

The conventional cephalometric analysis (two-dimensional) considers the FH plane as the horizontal plane. Based on two-dimensional to three-dimensional cephalometric analysis, the reliability and reproducibility of CBCT have been the major challenges. To achieve consistency and reproducibility, our CBCT images were calibrated based on the following rules: (1) the sagittal plane separates the skull into the left and right sides at the orbital area; (2) the horizontal plane is parallel to the FH plane in 3D images; and (3) the coronal plane is perpendicular to the two planes mentioned earlier.

Owing to factors such as mandibular development and muscular attachment, the MF margin can be irregular. The MF front exhibits a prominent bone, the top of which presents a projection ridge, which is anatomically known as the Li. The Li is located on the medial aspect of the ramus and is a tongue-shaped bony projection above the MF. The Li is also a vital anatomical site during dental local anesthetic injection to achieve an IAN block [4]. While the sphenomandibular ligament is attached to the Li, this attachment is likely to affect the Li shape [1, 5]. Using dry adult human mandibles, Tuli et al. [6] categorized the Li into four groups: triangular shape, truncated shape, nodular shape, and assimilated shape. Accounting for 68% of the mandibles, the triangular shape is the most commonly observed group.

Because the Li cannot directly be seen, surgeons have been seeking a suitable reference point to serve as a guideline in the osteotomy. This is to avoid injury to the inferior alveolar neurovascular bundle, which could cause massive hemorrhage during surgery and postoperative lower lip numbness. Surgeons discovered that a bony tubercle or prominence, known as antilingula, appears at the opposite side of the Li to the lateral aspect of the ramus. However, studies [7–12] have demonstrated that considerable uncertainties exist concerning the relation between the positions of the antilingula and the Li and MF. Therefore, when the antilingula serves as the reference point to locate the Li during surgery, the values measured using 3D computed tomography should be adopted for safety concerns.

SSRO is performed at the medial side of the ramus and was originally described by Trauner and Obwegeser [13]. It can be used for mandibular advancement in case of mandibular deficiency or setback for mandibular prognathism. The horizontal procedure for SSRO is performed above the Li and travels along the EOR, ending at the mandibular molar region, thus leaving sufficient space between the IOR and EOR to avoid unfavorable mandibular fracture, which could be caused by bad split. This procedure then continues on the medial side of the EOR and moves on to the buccal bone at the mandibular molar region. Therefore, measuring the distances (Li-EOR, Li-IOR, EOR-PBR, IOR-PBR) and ratios (Li-EOR/ EOR-PBR and Li-IOR/ IOR-PBR) is very important in surgical procedures.

While Yeh et al. [4] reported that the Li-EOR/ EOR-PBR ratio was 0.52, and Sekerci and Sisman [14] estimated the Li-EOR/ EOR-PBR ratio to be 0.559. In addition, the Li-EOR/ EOR-PBR ratio was 0.523 according to Senel et al. [2]. The results of this study revealed that the Li-EOR/ EOR-PBR ratio was 55.8%. The Li-EOR/ EOR-PBR ratio among men was 55.7% while that among women was 55.9%. The Li-EOR/ EOR-PBR ratios in Class I, Class II, and Class III were 55.1%, 54.7%, and 57.4%, respectively. The results were similar to those obtained in the aforementioned studies, confirming that the Li is located at a position slightly posterior to the 1/2 anteroposterior diameter of the ramus from the EOR viewpoint.

When the IOR served as the reference point for Li location, the Li-IOR/IOR-PBR ratio was 45.2% overall. The average values of Li-IOR/IOR-PBR ratios in men and women were 45.3% and 45.4%, respectively. The Li-IOR/IOR-PBR ratios in Class I, Class II, and Class III reached 44.7%, 45.3%, and 46.2%, respectively. Specifically, the Li was slightly anterior to the 1/2 anteroposterior diameter of the ramus from the IOR viewpoint. When SSOR was conducted with a short horizontal cut, we suggested using the EOR as the reference point and making the incision to approximately 60% of the ramus anteroposterior diameter. However, from the IOR viewpoint, the incision should be made to roughly 50% of the ramus anteroposterior diameter. This is because at this position, a bad split at the Li could be avoided. In some cases, the IOR could be less distinct than the EOR. In our study, the average distance between the EOR and IOR was 6.5 mm and we could determine the position of the IOR.

Sekerci and Sisman [14] reported that the Li-Sm was 15.32 mm and Li-IBR was 33.43 mm, resulting in a Li vertical ratio (Li-Sm/Sm-IBR) of 0.341. Senel reported that the Li-Sm was 18.1 mm and Li-IBR was 38.8 mm; therefore, a value of 0.321 was attained for the Li vertical ratio. The Li vertical ratio in this study was 39.1%. In addition, the Li vertical ratio from the Sm aspect was 38% in men and 39.7% in women. This ratio in male participants was significantly close to Sm, indicating a higher Li position. The vertical ratios of Li were 38.7% in Class I, 39.8% in Class II, and 39% in Class III. In summary, the vertical Li positions in men, Class I, and Class III were significantly close to the Sm. When Sm served as the viewpoint for Li location at the vertical diameter of the ramus, osteotomy should be made through 1/3 of the vertical diameter of the ramus from Sm. At this point, osteotomy should be conducted above the Li and should not cause injury to the inferior alveolar neurovascular bundle.

Sekerci and Sisman [14] reported a Li-MF vertical distance of 7.9 mm, whereas the distance measured by Senel was 7.8 mm. Similarly, the measured distance in the present study was 8.02 mm. This finding reminds surgeons that when performing IVRO, the Li-MF must be carefully considered since the Li cannot be directly observed. A distance between the Li and MF must be retained for a 10 mm vertical cut, and then continuous vertical osteotomy or slightly forward oblique osteotomy can be employed to prevent injury to the inferior alveolar neurovascular bundle.

EOR, IOR, and Sm could all be seen and served as surgical landmarks during SSRO. In Pearson's correlation test, we did not find Li-Sm significant correlation to Li-EOR and Li-IOR. This indicated that the Li-Sm distance cannot be predetermined by simply using the Li-EOR and Li-IOR. Li-hMF was positive, yet weakly correlated with Li-EOR and Li-IOR, indicating a longer length in Li-EOR or Li-IOR and Li-hMF. This means that the lingula protruded more from the medial aspect of the ramus and was more touchable during SSRO. Therefore, the inferior alveolar neurovascular bundle could be avoided for safety concerns. Notwithstanding, our findings presented a good procedure for SSRO. However, research landmarks were orthogonal to a projective plane (ramus). In further study, the nonorthogonal projections could provide useful information.

## 5. Conclusion

Using the Li as a reference point to measure the distances, there were no significant differences between the right and left sides. The Li-EOR, Li-PBR, and Li-IBR were significantly longer in men than in women. Additionally, the Li-hMF and Li-vMF were significantly longer in men than in women. The results demonstrated that the Li was longer and more protruding in men than in women. Of the three types of skeletal patterns (Class I, Class II, and Class III), Class III (Li-hMF) was the significantly largest.

## Figures and Tables

**Table 1:** Distance of ramal landmarks related to lingula with hemiarch comparisons.

Variables	Total patients		Right side		Left side		Right/Left comparison	
	Mean	SD	Mean	SD	Mean	SD	*p* value	
Li-EOR	19.21	3.02	19.23	2.49	19.20	3.49	0.954	─
Li-IOR	12.66	2.22	12.84	2.30	12.47	2.15	0.319	─
Li-PBR	15.22	2.02	15.25	1.96	15.19	2.09	0.860	─
Li-vMF	8.07	2.39	8.20	2.13	7.95	2.64	0.526	─
Li-Sm	20.04	3.16	20.51	3.39	19.57	2.86	0.073	─
Li-IBR	31.20	3.81	31.26	3.80	31.14	3.85	0.850	─
Li-hMF	2.27	1.44	2.05	1.36	2.49	1.49	0.063	─

^*∗*^: significant, *p* < 0.05. ─: not significant.

**Table 2:** Distance of ramal landmarks related to lingula with their gender comparisons.

Variables	Male		Female		Gender comparison		
	Mean	SD	Mean	SD	*p* value		
Li-EOR	19.99	2.84	18.85	3.05	0.032	^*∗*^	Male > female
Li-IOR	13.20	2.58	12.40	2.00	0.069	─	
Li-PBR	15.93	1.75	14.89	2.05	0.002	^*∗*^	Male > female
Li-vMF	8.73	2.04	7.76	2.49	0.015	^*∗*^	Male > female
Li-Sm	20.14	3.38	19.99	3.07	0.783	─	
Li-IBR	32.91	3.79	30.40	3.57	<0.001	^*∗*^	Male > female
Li-hMF	2.81	1.80	2.01	1.16	0.008	^*∗*^	Male > female

^*∗*^: significant, *p* < 0.05. ─: not significant.

**Table 3:** Distance of ramal landmarks related to lingula with their skeletal comparisons.

Variables	Class I		Class II		Class III		Intergroup comparison			
	Mean	SD	Mean	SD	Mean	SD	*F*	*p* value		Tukey HSD postcomparison
Li-EOR	18.86	3.51	18.71	2.48	20.00	2.78	2.678	0.072	─	
Li-IOR	12.45	1.88	12.83	2.76	12.73	2.07	0.372	0.690	─	
Li-PBR	15.36	2.04	15.51	1.92	14.83	2.05	1.476	0.232	─	
Li-vMF	8.24	1.93	7.83	2.64	8.11	2.62	0.345	0.709	─	
Li-Sm	19.74	3.16	20.72	3.42	19.77	2.91	1.378	0.255	─	
Li-IBR	31.28	3.71	31.40	3.86	30.95	3.94	0.176	0.839	─	
Li-hMF	2.12	1.27	1.80	1.44	2.81	1.46	6.537	0.002	^*∗*^	Class III > class I, Class III > Class II

^*∗*^: significant, *p* < 0.05. ─: not significant.

**Table 4:** Pearson test in the distance of ramal landmarks related to lingula.

Variables	Li-EOR	Li-IOR	Li-PBR	Li-vMF	Li-Sm	Li-IBR	Li-hMF
Li-EOR	1	0.610^*∗*^	0.012	0.013	−0.031	0.170^*∗*^	0.278^*∗*^
Li-IOR	0.610^*∗*^	1	−0.085	−0.236^*∗*^	0.063	−0.0198	0.389^*∗*^
Li-PBR	0.0125	−0.085	1	0.191^*∗*^	0.161	0.338^*∗*^	−0.205^*∗*^
Li-vMF	0.013	−0.236^*∗*^	0.191^*∗*^	1	−0.197^*∗*^	0.329^*∗*^	0.158
Li-Sm	−0.031	0.063	0.161	−0.197^*∗*^	1	−0.091	−0.025
Li-IBR	0.170^*∗*^	−0.020	0.338^*∗*^	0.329^*∗*^	−0.091	1	−0.235^*∗*^
Li-hMF	0.278^*∗*^	0.389^*∗*^	−0.205^*∗*^	0.158	−0.025	−0.235^*∗*^	1

^*∗*^Statistically significant, *p* < 0.05.

## Data Availability

The data used to support the findings of this study are available from the corresponding author upon request.

## References

[1] Drake R. L., Vogl A. W., Mitchell A. W. (2010). *Head and Neck: Gray’s Anatomy for Students*.

[2] Senel B., Ozkan A., Altug H. A. (2015). Morphological evaluation of the mandibular lingula using cone-beam computed tomography. *Folia Morphologica*.

[3] Riedel R. A. (1950). Esthetics and its relation to orthodontic therapy. *The Angle Orthodontist*.

[4] Yeh A. Y. E., Finn B. P., Jones R. H. B., Goss A. N. (2018). The variable position of the inferior alveolar nerve (IAN) in the mandibular ramus: a computed tomography (CT) study. *Surgical and Radiologic Anatomy*.

[5] Jansisyanont P., Apinhasmit W., Chompoopong S. (2009). Shape, height, and location of the lingula for sagittal ramus osteotomy in Thais. *Clinical Anatomy*.

[6] Tuli A., Choudhry R., Choudhry S., Raheja S., Agarwal S. (2000). Variation in shape of the lingula in the adult human mandible. *Journal of Anatomy*.

[7] Park K.-R., Kim S.-Y., Kim G.-J., Park H.-S., Jung Y.-S. (2014). Anatomic study to determine a safe surgical reference point for mandibular ramus osteotomy. *Journal of Cranio-Maxillofacial Surgery*.

[8] Monnazzi M. S., Passeri L. A., Gabrielli M. F. R., Bolini P. D. A., de Carvalho W. R. S., da Costa Machado H. (2012). Anatomic study of the mandibular foramen, lingula and antilingula in dry mandibles, and its statistical relationship between the true lingula and the antilingula. *International Journal of Oral and Maxillofacial Surgery*.

[9] Aziz S. R., Dorfman B. J., Ziccardi V. B., Janal M. (2007). Accuracy of using the antilingula as a sole determinant of vertical ramus osteotomy position. *Journal of Oral and Maxillofacial Surgery*.

[10] Park J. H., Jung H. D., Kim H. J., Jung Y. S. (2018). Anatomical study of the location of the antilingula, lingula, and mandibular foramen for vertical ramus osteotomy. *Maxillofacial Plastic and Reconstructive Surgery*.

[11] Apinhasmit W., Chompoopong S., Jansisyanont P. (2011). The study of position of antilingula, midwaist of mandibular ramus and midpoint between coronoid process and gonion in relation to lingula of 92 Thai dried mandibles as potential surgical landmarks for vertical ramus osteotomy. *Surgical and Radiologic Anatomy*.

[12] Pogrel M. A., Schmidt B. L., Ammar A. (1995). The presence of the antilingula and its relationship to the true lingula. *British Journal of Oral and Maxillofacial Surgery*.

[13] Trauner R., Obwegeser H. (1957). The surgical correction of mandibular prognathism and retrognathia with consideration of genioplasty. I. Surgical procedures to correct mandibular prognathism and reshaping of the chin. *Oral Surgery, Oral Medicine, Oral Pathology*.

[14] Sekerci A. E., Sisman Y. (2014). Cone-beam computed tomography analysis of the shape, height, and location of the mandibular lingula. *Surgical and Radiologic Anatomy*.

